# Managing Ammonia Emissions from Dairy Cows by Amending Slurry with Alum or Zeolite or by Diet Modification

**DOI:** 10.1100/tsw.2001.100

**Published:** 2001-10-27

**Authors:** John J. Meisinger, Alan M. Lefcourt, Jo Ann S. Van Kessel, Victor Wilkerson

**Affiliations:** ^1^ USDA-ARS, Animal Manure and By-Product Laboratory, Building 163F BARC-East, Beltsville, MD 20705, USA; ^2^ USDA-ARS, Animal and Natural Resources Institute, BARC-East, Beltsville, MD 20705, USA

**Keywords:** alum, ammonia emissions, ammonia volatilization, dairy slurry, manure management, zeolite

## Abstract

Animal agriculture is a significant source of atmospheric ammonia. Ammonia (NH3) volatilization represents a loss of plant available N to the farmer and a potential contributor to eutrophication in low-nitrogen input ecosystems. This research evaluated on-farm slurry treatments of alum or zeolite and compared three diets for lactating dairy cows in their effectiveness to reduce NH3 emissions. NH3 emissions were compared using a group of mobile wind tunnels. The addition of 2.5% alum or 6.25% zeolite to barn-stored dairy slurry reduced NH3 volatilization by 60% and 55%, respectively, compared to untreated slurry. The alum conserved NH3 by acidifying the slurry to below pH 5, while the zeolite conserved ammonia by lowering the solution-phase nitrogen through cation exchange. The use of alum or zeolite also reduced soluble phosphorus in the slurry. NH3 loss from fresh manure collected from lactating dairy cows was not affected by three diets containing the same level of crude protein but differing in forage source (orchardgrass silage vs. alfalfa silage) or neutral detergent fiber (NDF) content (30% vs. 35% NDF). NH3 losses from the freshly excreted manures occurred very rapidly and included the urea component plus some unidentified labile organic nitrogen sources. NH3 conservation strategies for fresh manures will have to be active within the first few hours after excretion in order to be most effective. The use of alum or zeolites as an on-farm amendment to dairy slurry offers the potential for significantly reducing NH3 emissions.

## INTRODUCTION

Ammonia ($NH_3$) volatilization from farm manure is a major source of N loss to the environment. The lost $NH_3$ affects farm economics by causing farmers to purchase N fertilizers for crops, and contributes to eutrophication in low-N input ecosystems through atmospheric transport and deposition[1,2]. Atmospheric $NH_3$ of agricultural origin has been implicated in widespread damage to natural ecosystems in Europe[2,3]. In addition, atmospheric $NH_3$ combines with sulfur-containing by-products of combustion to form small particulates (PM 2.5), which can cause respiratory disease.

Agriculture is the major source of $NH_3$ emissions to the atmosphere, contributing an estimated 90% of total $NH_3$ emissions in the U.S.[4] and in Western Europe[5,6,7]. Within the agriculture sector, the largest losses are thought to occur during land application (35–45%), followed by losses during housing (30–35%), grazing (10–25%), and then storage (5–15%)[5, 8, 9]. Most efforts to reduce $NH_3$, which have highlighted the benefits of immediate soil incorporation[11,12], have focused on land application[7,10,11,12]. Less effort has been devoted to examining $NH_3$ abatement approaches applicable to the manure management system or to the cow. This paper will therefore report $NH_3$ loss studies focusing on farm-level manure treatment and on animal diet.

$NH_3$ volatilization from manure management systems requires conversion of ammonium-N ($NH_4$-N) to dissolved $NH_3$ gas and gaseous exchange to the atmosphere. $NH_3$ controls focus on reducing the pH, which reduces the quantities of dissolved $NH_3$ gas, and on limiting gas exchange. Chemical measures, which reduce pH, include acidifying liquid manures with mineral acids[13] or adding alum to solid manure[14]. Physical measures that reduce gas exchange include reduction of particulates in manure slurry, covering of manure storage facilities, encouragement of crust formation on manure surfaces, and addition of agents that sequester ammonia[5,15]. The high cost of modifying manure storage facilities such as lagoons has prompted renewed interest in some form of on-farm manure treatment to reduce $NH_3$ emissions.

Diet can impact potential $NH_3$ loss by altering the quantity of excreted N, or the partitioning of N between urine and feces. The primary source for $NH_3$ loss is urinary N; thus, feeding strategies that reduce urinary N should reduce $NH_3$ losses[16,17,18]. One possible approach for cattle is to improve the rumen synchronization of carbohydrate and protein degradation to conserve $NH_3$ in microbial protein and thereby lower N removed by the kidneys[19]. This approach has been demonstrated to reduce N excretion from cattle[20].

The objective of this research was to evaluate two on-farm slurry treatments and three diets for lactating dairy cows and their effectiveness in reducing $NH_3$ emissions.

## EXPERIMENTAL METHODS

### Slurry Amendment Studies

The barn-stored slurry was collected from the holding pit of a 100-cow, free-stall barn at the Beltsville Agriculture Research Center after one hour of agitation. The manure collection system was a mechanical scrapper that continuously moved manure into the holding pit. The cows were fed a total mixed ration (TMR) based on corn or alfalfa silage, corn grain, and protein supplement. Bedding consisted of a small amount of sawdust, which produced a slurry with a 9–11% dry matter content. Bulk samples of the slurry were collected and were treated with the following: nothing (control slurry), 2.5% by weight alum (granular $Al_2(SO_4)_3$ $*14H_2O$), or 6.25% by weight zeolite (200-mesh clinoptilolite from Nicole Mt., $NH_4$-N exch. cap. 1.8 – 2.0 meq. $g^{-1}$). Preliminary trials indicated that these levels of amendments provided optimal ammonia-sequestering capacity[21]. Alum is commonly used as a flocculent in sewage treatment, and has been shown to lower ammonia emissions from poultry litter[14]. Zeolites are silicate clay minerals widely available in the western U.S. The zeolite is a natural cation exchange media used both in aquaculture to reduce $NH_3$ and in pet products such as kitty litter.

Subsamples of all treated and control slurries were frozen and were chemically analyzed for various N species (see below), phosphorus (P), pH, and aluminum (Al), as described in detail in Lefcourt and Meisinger[21]. Briefly, the analyses consisted of the following: total Kjeldahl N (TKN) and P (TKP) by block digestion and colorimetric $NH_4$-N analysis using the Bertholet reaction (Technicon Ind. Meth. 334-74W/B), or colorimetric analysis for orthophosphate-P by the molybdate ascorbic acid method[22], moisture content by drying at 100°C, pH by directly inserting a glass-calmel combination electrode, soluble orthophosphate-P by the molybdate ascorbic acid[22], and Al by atomic adsorption spectrophotometry. Slurry subsamples were also extracted with water and 1 *M* KCl; the filtered extracts were analyzed for $NH_4$-N, *ortho*-P, pH, and Al as described above. These extracts allowed chemical characterization of each slurries' solution phase (water extract) and the solution plus exchangeable $NH_4$-N phase (1 *M* KCl). Differences in ammonia concentrations in 1 *M* KCl and water extracts represent exchangeable $NH_4$-N.

Ammonia volatilization from the barn-stored slurries was assessed using six small wind tunnels as described in detail by Meisinger et al.[23]. Each tunnel consists of two components, an inverted U-shaped canopy (2.0 × 0.5 m) and an attached metal plenum that contains a variable speed fan, a six-spoked cross-section air sampler, and an anemometer to continuously monitor wind speed. Tunnel temperatures ranged between 9 and 14°C, and wind speeds were set at 0.5 m $sec^{-1}$. Gas scrubbing bottles containing 2 m*M* $H_3$ $PO_4$ along with small vacuum pumps were used to trap $NH_3$ from the air entering and leaving each canopy. Controlled loss-and-recovery experiments under similar conditions reported[23] recoveries of 104 ± 6%. For each wind tunnel, the slurry was poured into two fiberglass trays (each 46 × 66 cm) to a depth of about 1.3 cm, and the trays were placed under the canopy. The $H_3$ $PO_4$ scrubbers were changed three or four times daily over 7 days; scrubbers were brought to volume, and refrigerated subsamples were analyzed for $NH_4$-N as described above. Results are expressed as cumulative $NH_3$ loss as a percentage of the slurry TKN added to each tunnel.

### *Diet Studies*

The diets were derived from a companion trial[24] evaluating the response of first-lactation Holstein cows to forage sources with different fiber contents. Two cows per ration were fed one of three TMR containing the same level of crude protein but with different forage sources. Diet I used orchardgrass silage (OS), while diet II used alfalfa silage (AS). Both diets were formulated to contain 30% neutral detergent fiber (NDF). In diet III, OS replaced AS on a weight basis that resulted in a diet with 35% NDF. It was hypothesized that these differences in fiber source might affect $NH_3$ volatilization because fiber source, or content, can have a significant impact on ruminal fermentation with possible consequences in the quantities of N leaving the rumen as microbial protein vs. N leaving the rumen as excess ammonia, which leads to increased urinary N.

Urine was collected from each cow over 24 h in sterilized jugs using bladder catheters, and feces were collected on stainless steel trays. The fresh samples of urine and feces were analyzed for TKN by block digestion and colormetric analysis as described above. Urine was further analyzed for the following: $NH_4$-N by steam distillation with MgO, urea-N using the Technicon Ind. Meth. 339-01, and pH using a glass-calmel electrode directly inserted in the sample.

The fresh urine and feces from each cow were combined in the excreted proportion and then mixed. The manure was poured into two fiberglass trays that were placed in the canopy of the wind tunnels as described above. The $NH_3$ volatilization studies were continued over seven days, with gas scrubber samples collected three or four times daily and analyzed for $NH_4$-N as described above. Temperatures ranged between 13 and 17°C, and tunnel wind speeds were maintained at 0.5 m $sec^{-1}$. Results are expressed as cumulative $NH_3$ loss as a percentage of the urinary TKN added to each tunnel.

## RESULTS AND DISCUSSION

### Slurry Amendment Studies

Cumulative $NH_3$ loss for the unamended barn-stored slurry (Fig. 1) shows a very rapid initial rate of loss, with about 65% of the total loss occurring within 24 h. Beyond 24 h the losses decreased to a nearly linear rate of about 1.5% of the slurry TKN per day. The total $NH_3$ loss from the control slurry was about 15% of the slurry TKN. This loss is less than that expected from freshly excreted manure, because the slurry had been exposed to the atmosphere for several hours in the free-stall barn and had been agitated in the holding pit for 1 h before collection. $NH_3$ losses from either 2.5% alum or 6.25% zeolite were negligible after 12 h, and total losses amounted to about 5% of the slurry TKN with alum and about 7% of the TKN for zeolite (Fig 1). The losses from the alum treatment before 12 h are attributed to degassing of previously dissolved $NH_3$ gas and the fact that the alum reacted slowly with the slurry at the cool temperatures (9 – 14°C) of this study. Thus, compared to the controls, the alum reduced $NH_3$ losses about 60%, while zeolite reduced losses about 55%.

The mode of action of alum is quite different than zeolite. The alum preserved $NH_3$ by acidifying the slurry. The untreated slurry had an initial pH of 7.7, while the alum-treated slurry had a pH of 4.7 (Table 1). The percentage of the total solution $NH_4$-N plus $NH_3$-N that is $NH_3$ gas is about 6% at pH 8 and about 0.006% at pH 5[25]. Acidification is therefore a potent method to conserve $NH_3$. The zeolite, on the other hand, preserved $NH_3$ by reducing the slurry-dissolved $NH_4$-N (which is in equilibrium with dissolved $NH_3$ gas) by absorbing the $NH_4$-N on the zeolite exchange sites (Table 1). For example, the zeolite-treated slurry held nearly 25% of the slurry TKN on exchange sites compared to only about 5% in exchangeable form in the unamended slurry. Others have also shown zeolite to have excellent $NH_3$-absorbent properties[26]. These results demonstrate that acidification with alum and sequestering $NH_4$-N on zeolite exchange sites are both effective methods to reduce $NH_3$ losses from dairy slurry.

The alum and zeolite amendments had additional effects on the raw slurry in addition to conserving $NH_3$. Both amendments resulted in reductions in water-soluble P (Table 1). The alum reduced soluble P to only 1% of the TKP, compared to 35% in the control. This reduction occurred because soluble Al reacts with P to form relatively insoluble Al- phosphate intermediates

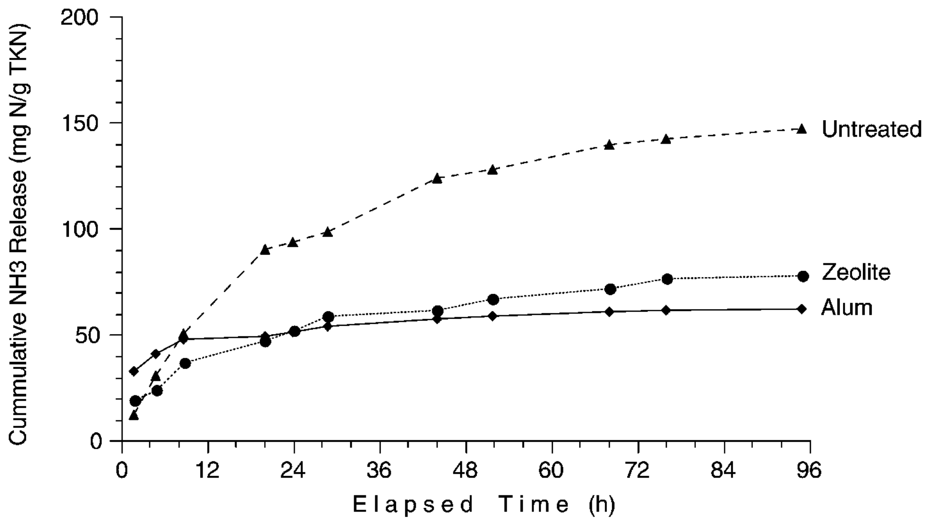

**FIGURE 1**

**TABLE 1**

| Slurry Treatment | pH | NH4-N in KCl and water extracts, expressed as % of slurry TKN | | | Water Soluble P Conc., as % of slurry TKP | Water soluble Al, ug/g manure |
|---|---|---|---|---|---|---|
| | | 1M KCl | Water | Exch. NH4-N | | |
| Control | 7.7 | 52% | 47% | 5% | 35% | 10 |
| 2.5% alum | 4.7 | 51% | 47% | 4% | 1% | 550 |
| 6.25% zeolite | 7.8 | 55% | 31% | 24% | 14% | 50 |

that form the reaction sequence from water soluble P to the insoluble variscite-like compounds[27]. The reduction in soluble P from zeolites was unexpected and was likely due to P adsorption on the zeolite mineral and/or to the formation of Ca- or Mg-phosphates mineral intermediates. Alumino-silicate minerals have been shown to retain P through both adsorption and precipitation mechanisms[27].

The on-farm treatment of dairy slurry with alum has drawbacks. The alum adds Al, which can increase the level of water-soluble Al in the slurry (Table 1). Excessive levels of soluble Al in slurries could add a soil acidity management element to farm nutrient management, but the specifics of this acidity management will depend on the farms' soil chemical properties, the crop rotation, and the liming program of the individual farm. The zeolite did not substantially increase the level of soluble Al. Another potential problem with on-farm treatment with alum is the physical effect that the alum treatment may have on the slurry. The addition of alum produced a marked effervescence from the slurry that caused handling difficulties. Alum can also lead to flocculation and separation of solids from the slurry. This flocculation effect could be beneficial in solid-separation manure management systems, or it could lead to handling difficulties in other systems. No physical difficulties or handling problems were encountered with the zeolite treatment.

These studies demonstrate that $NH_3$ can be conserved with on-farm treatment of barn-stored dairy slurry with alum or zeolite. Both of these treatments also give secondary benefits in reducing soluble P, although the increased soluble Al from alum may require more attention to acidity management. Dry alum was used in this study. Liquid alum with a lower effective pH is commercially available, and its use would reduce the likelihood of adding excess alum during treatment. Alum treatment can also lead to effervescence and physical handling problems for liquid manure management systems. Based on current costs for materials only, either the liquid alum treatment or the zeolite treatment would cost between \$0.50 and \$1.00 cow$^{-1}$ day$^{-1}$. More precise cost estimates cannot be made without better knowledge of the equipment needs for adding the amendments and the economies of scale that would lower material costs.

## Diet Studies

The three diets significantly affected the cows' production parameters[24]. Dry matter (DM) intakes and milk yields were not different for diet I (OS) vs. diet II (AS) with both having 30% NDF, but DM intakes and milk yields were lower with diet III (35% NDF), amounting to 19 kg less DM intake day$^{-1}$ and 27 kg less milk day$^{-1}$. Cows fed diet I gained the most weight during the 11-week study (49 kg), while diet-II cows gained an intermediate amount (39 kg), and diet-III cows gained the least weight (30 kg). The N chemistry of the feces, urine, and manure was not significantly different for these diets (Table 2), despite the differences in forage source. However, it should be recalled that these diets were balanced to supply the same level of crude protein. The proportion of the manure derived from urinary N was also not different for the three diets (Table 2). Apparently these diets did not alter the patterns of rumen N utilization enough to change the proportion of N excreted in the urine vs. feces.

The wind tunnel $NH_3$ volatilization studies from the fresh manures derived from these diets (Fig. 2) show little or no effect of diet on $NH_3$ loss. The total $NH_3$ loss, expressed as a percentage of the urinary TKN for each diet, was the following: 104% for diet I (OS at 30% NDF), 105% for diet II (AS at 30% NDF), and 97% for diet III (OS at 35% NDF), with a standard error of the mean of about 9%. The reason for the lack of dietary effect on $NH_3$ loss is likely due to the lack of difference in urine vs. fecal N excretions, the similar percentage of urine in each manure, and the similar pH of all the manures (Table 2). Thus, the basic chemistry driving $NH_3$ losses from these fresh manures (urea and $NH_4$-N content and pH) was similar for all the diets.

Although the total $NH_3$ losses among diets were similar, there are two noteworthy features of Fig. 2 that illustrate important points for $NH_3$ management of fresh dairy manures. The first point is that $NH_3$ losses began immediately after exposing the fresh manures to the atmosphere; in fact, almost all of the total loss occurred during the first 48 h after exposure. Losses were small after 48 h due to crust formation on the manures, which limited gas exchange. The second point is that the total N loss was roughly equal to the TKN content of the urine. The freshly

**TABLE 2**

| Diet Identification and Description<br>OS=Orchgr. Silage<br>AS= Alfalfa Silage<br>NDF=Neutral Detergent Fiber | Feces | Urine Characteristics | | Manure Characteristics | |
|---|---|---|---|---|---|
| | T K N<br>g N | $NH_4$-N + Urea-N<br>g N | T K N<br>g N | Urinary T K N as % of Manure T K N | Manure pH |
| I: OS, 30%NDF | 208 | 55 | 66 | 24 % | 7.9 |
| II: AS, 30%NDF | 238 | 70 | 77 | 24 % | 8.2 |
| III: OS, 35%NDF | 222 | 77 | 85 | 28 % | 7.9 |
| Std. Error Mean : | 17 | 11 | 11 | 3 % | 0.2 |

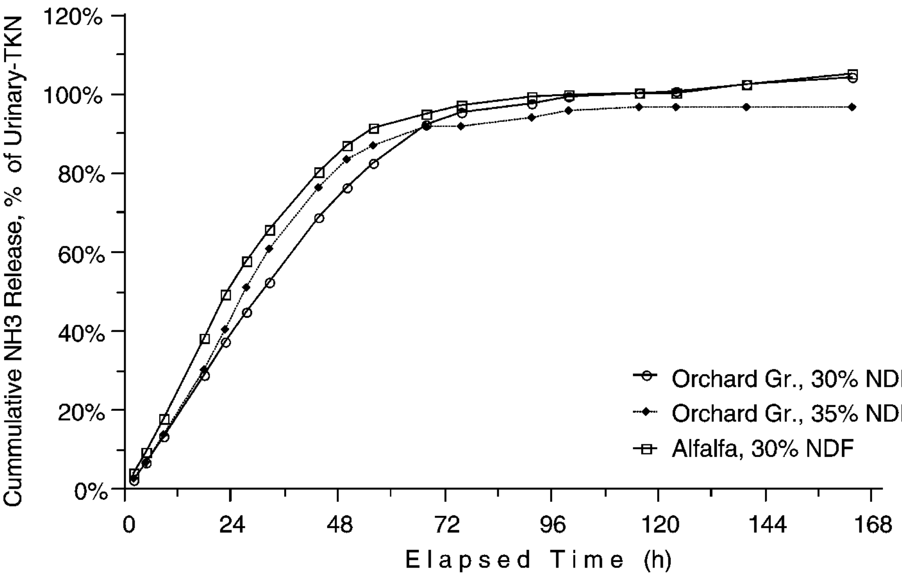

**FIGURE 2**

excreted urine contained about 88% of its TKN as urea plus $NH_4$-N (Table 2), which means that a portion of the organic N compounds in the urine and/or feces were also highly labile and vulnerable to $NH_3$ volatilization. These other labile organic N compounds may be derived from methylamines or other organic N forms that can readily hydrolyze and release $NH_3$. Muck and Richards[28] postulated that under warm conditions significant quantities of non-urea organic N can be ammonified within 24 h of excretion.

## CONCLUSIONS

Agriculture is a significant source of atmospheric $NH_3$ and PM 2.5. $NH_3$ volatilization represents a loss of plant available N to the farmer and contributes to eutrophication in low-N input ecosystems. Urinary N is the main source of this $NH_3$ loss. The major farm components contributing to $NH_3$ loss are diet formulation, housing and manure management, grazing, manure storage, and land application. This research evaluated two on-farm slurry treatments and three diets for lactating dairy cows in their effectiveness to reduce $NH_3$ emissions. The addition of 2.5% alum or 6.25% zeolite to barn-stored dairy slurry reduced $NH_3$ volatilization by 60% and 55%, respectively, compared to untreated slurry. The alum conserved $NH_3$ by acidifying the slurry to below pH 5, while the zeolite conserved $NH_3$ by lowering the solution phase N through cation exchange. The use of alum or zeolite also reduced soluble P in the slurry. Alum must be carefully managed to minimize effervescence and avoid high concentrations of soluble Al in the slurry. Zeolite had no physical or chemical drawbacks. $NH_3$ loss from fresh manure collected from lactating dairy cows was not affected by three iso-protein diets which were formulated from either orchardgrass silage or alfalfa silage at 30% NDF, or an orchardgrass silage diet with 35% NDF. $NH_3$ losses from fresh manures occurred very rapidly and included the urinary-urea component plus some unidentified labile organic N sources that were rapidly ammonified. Management procedures designed to prevent $NH_3$ losses from fresh manures will have to be active within the first few hours after excretion in order to be most effective. The use of alum or zeolites as an on-farm amendment to barn-stored dairy slurry offers the potential for significantly reducing $NH_3$ emissions.

| | | |
|---|---|---|
| Received: | July | 18, 2001 |
| Revised: | September | 10, 2001 |
| Accepted: | September | 12, 2001 |
| Published: | October | 27, 2001 |
